# Exploring the Role of *Lycium barbarum* Polysaccharide in Corneal Injury Repair and Investigating the Relevant Mechanisms through In Vivo and In Vitro Experiments

**DOI:** 10.3390/molecules29010049

**Published:** 2023-12-20

**Authors:** Qian Liu, Yi Nan, Yifan Yang, Xiangyang Li, Wenjie Jiang, Taiqiang Jiao, Jiaqing Li, Xusheng Jia, Mengyi Ye, Yang Niu, Ling Yuan

**Affiliations:** 1 School of Pharmacy, Ningxia Medical University, Yinchuan 750004, China; 2 Key Laboratory of Ningxia Ethnomedicine Modernization, Ministry of Education, Ningxia Medical University, Yinchuan 750004, China; 2020011064@nxmu.edu.cn (Q.L.); 20080011@nxmu.edu.cn (Y.N.); 20210410191@nxmu.edu.cn (X.L.); 20210910213@nxmu.edu.cn (W.J.); 20220410205@nxmu.edu.cn (T.J.); 20220410222@nxmu.edu.cn (J.L.); 220220903@nxmu.edu.cn (X.J.); 3 College of Clinical Medicine, Ningxia Medical University, Yinchuan 750004, China; 4 School of Traditional Chinese Medicine, Ningxia Medical University, Yinchuan 750004, China; 20210910215@nxmu.edu.cn (Y.Y.); yexiaomiao1991@163.com (M.Y.)

**Keywords:** corneal injury, *Lycium barbarum* polysaccharides, apoptosis, inflammation, fibrosis, IL17 signaling pathway

## Abstract

*Lycium barbarum* polysaccharide (LBP) is the main active component of Fructus Lycii, exhibiting various biological activities. This study aims to explore the protective effects of LBP on human corneal epithelial cells (HCEC) and a rat corneal injury model. Potential target points for LBP improving corneal injury repair were screened from public databases, and functional and pathway enrichment analyses of core targets were conducted using Gene Ontology (GO) and Kyoto Encyclopedia of Genes and Genomes (KEGG). Rat corneal alkali burns and HCEC oxidative stress injury models were established, and the results were validated through slit lamp examination, HE staining, TUNEL assay, immunofluorescence, CCK-8 assay, flow cytometry, scratch assay, and qRT-PCR methods. In the context of database retrieval, identification of 10 LBP monosaccharide components and 50 corneal injury repair-related targets was achieved. KEGG pathway analysis suggested that LBP might regulate the IL-17 and TNF signaling pathways through targets such as JUN, CASP3, and MMP9, thereby improving corneal damage. In vivo and in vitro experimental results indicated that LBP could reduce the increase of inflammation index scores (*p* < 0.05), inflammatory cell density (*p* < 0.01), TUNEL-positive cells (*p* < 0.01), corneal opacity scores (*p* < 0.01), and expression of corneal stromal fibrosis-related proteins α-SMA, FN, and COL (*p* < 0.01) caused by chemical damage to rat corneas. LBP inhibited oxidative stress-induced decreases in cell viability (*p* < 0.001) and migration healing ability (*p* < 0.01) in HCECs, reducing apoptosis rates (*p* < 0.001), ROS levels (*p* < 0.001), and the expression of inflammatory factors TNF-α and IL-6 (*p* < 0.01). qRT-PCR results demonstrated that LBP intervention decreased the mRNA levels of JUN, CASP3, and MMP9 in H_2_O_2_-induced alkaline-burned corneas and HCECs (*p* < 0.01).The integrated results from network pharmacology and validation experiments suggest that the inhibitory effects of LBP on apoptosis, inflammation, and fibrosis after corneal injury may be achieved through the suppression of the TNF and IL-17 signaling pathways mediated by JUN, CASP3, and MMP9.

## 1. Introduction

Fructus Lycii (FL), also known as the *Lycium barbarum* or wolfberry, is a plant native to China’s Ningxia region that can be used as both food and medicine. The first documentation of this herb can be traced back to the *Shennong Herbal Classics*, an ancient document of great significance in the field of herbal medicine. The *Compendium of Materia Medica* provides a comprehensive overview of the benefits of this substance, highlighting its ability to improve vision by nourishing the liver and kidneys. *Lycium barbarum* polysaccharides (LBP) are the primary bioactive components of *Lycium barbarum*, and exhibit numerous biological activities, such as inhibition of oxidative stress, anti-inflammatory responses, inhibition of tumor growth, regulation of lipid metabolism, and suppression of fibrosis. By inhibiting cell apoptosis, LBP protects human corneal epithelial cells (HCEC) [1]. LBP substantially reduces the increased apoptosis rate induced by H_2_O_2_ and the production of ROS while increasing the level of the antioxidant enzyme SOD [2]. To date, research on the inhibition of corneal fibrosis by LBP has been limited. In a corneal fibroblast injury model [3,4], the ophthalmology research team at the University of Hong Kong Li Ka Shing Faculty of Medicine found that pretreatment with LBP reduced the expression of fibrosis-related proteins, including α-smooth muscle actin (α-SMA), fibronectin (FN), and collagen (Col). LBP pretreatment also decreased the contraction and rigidity of hydrogels embedded with corneal fibroblasts, indicating that LBP can reduce corneal fibrotic scarring by inhibiting the differentiation, proliferation, and Extracellular matrix (ECM) secretion of corneal fibroblasts. Nonetheless, the precise mechanisms of its action remain unclear.

The cornea is a highly specialized, transparent, circular tissue composed of epithelial, anterior elastic, stromal, posterior elastic, and endothelial layers. It serves the dual purpose of protecting the contents of the eye and contributing to the optical system [5]. Due to its position at the front of the eye, the cornea is susceptible to a variety of external assaults and injuries. Over a million cases of ocular injuries occur annually in the United States [6]. It is estimated that approximately 20% of the population will experience ocular trauma during their lifetime. Inadequate restoration following corneal injury can result in corneal epithelial defects, stromal fibrotic scarring, vision impairment and, in extreme cases, corneal blindness [7]. Corneal scarring accounts for 2.4% of the world’s 253 million cases of blindness and visual impairment, making it the fourth leading cause of blindness overall [8,9]. There are currently no effective treatments for vision loss caused by corneal injuries. Steroid drugs such as dexamethasone (Dex) are commonly used, but their efficacy is limited and they frequently cause complications. Corneal transplantation is the only treatment option for corneal blindness, but it is accompanied by high surgical costs, a lack of corneal donors, potential infection risks, and rejection reactions, which place a significant emotional and financial burden on patients and significantly diminish their quality of life [10]. Therefore, the search for new pharmaceuticals and methods for preventing and treating corneal injuries is clinically significant.

Based on the etiology of corneal diseases, corneal injuries can be classified into five main classes: biological, physical, chemical, impact from other organs, and genetic factors. Chemical corneal injury is a prevalent form of corneal damage that can result in corneal epithelial defects, corneal inflammation, corneal opacification, and fibrotic scar formation. It functions as an exhaustive model for corneal injury research. In clinical contexts, corneal injuries are frequently accompanied by inflammation and oxidative stress [11,12]. During oxidative stress, excessive production of reactive oxygen species (ROS) can cause cell injury by targeting DNA, proteins, and intracellular pathways [13]. H_2_O_2_ is a common reactive oxygen species that can cause oxidative stress and inflammatory responses [14].

To explore the potential effects and specific mechanisms of LBP on in vivo and in vitro corneal injury models, we first utilized the emerging traditional Chinese medicine network pharmacology approach, based on recent advancements in network theory and systems biology, to predict and analyze key targets and pathways by which LBP may improve corneal injury. Then, we established corneal alkali burn and H_2_O_2_-induced HCEC cell injury models in rats and intervened with LBP. Observing the effects of LBP on in vivo corneal injury and ex vivo corneal epithelial cell injury, we investigated the underlying mechanisms. In addition, we validated the network pharmacology-predicted targets in biological processes in an effort to provide reference points for future research and clinical applications of LBP in the treatment of corneal injury.

## 2. Results

### 2.1. Target Screening and PPI Network Construction for the Potential Role of LBP in Corneal Injury Repair

**Figure 1:**
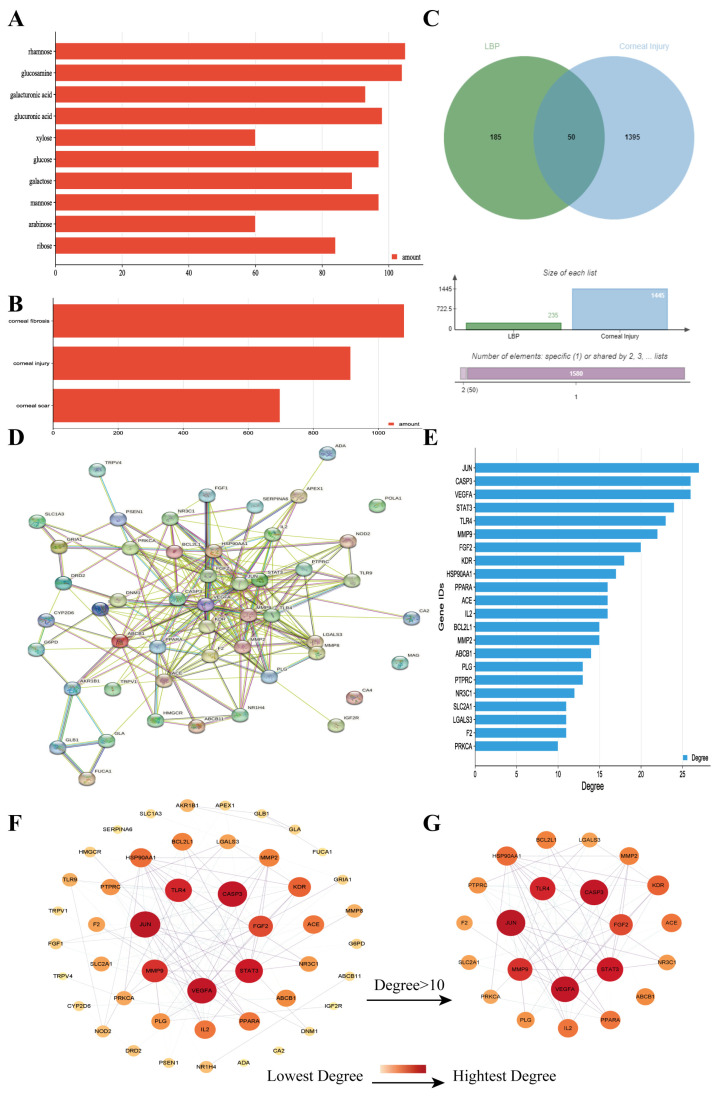
LBP improves corneal injury repair potential target screening and PPI network analysis. (**A**) Number of targets for each monosaccharide component of LBP; (**B**) number of disease-related targets; (**C**) Venn diagram of intersecting targets between LBP and corneal injury; (**D**) PPI network diagram of LBP treatment for corneal injury targets; (**E**) specificity values of core targets; (**F**) PPI network diagram sorted by degree value; (**G**) PPI network diagram of core targets with degree values > 10.

Through a literature review, the main monosaccharide components of LBP include arabinose, glucose, galactose, mannose, xylose, rhamnose, glucuronic acid, galacturonic acid, ribose, and glucosamine [15,16]. Using the TCMSP and PubChem databases, various monosaccharide components were searched, and their chemical structures, AlogP values, OB values, linear notations, and other relevant information are listed in Table 1. AlogP values represent the partition coefficient between octanol and water, and all ten major monosaccharide components of LBP have negative AlogP values, indicating good water solubility. An OB value greater than or equal to 30% is a threshold for assessing whether a drug has a high oral availability. Predictions in the Swisstarget database revealed the associated target genes for various monosaccharide components of LBP, showing that, among them, ribose had the highest number of related targets, totaling 105 genes (Figure 1A). After removing the duplicate target gene information for each monosaccharide component, a total of 235 LBP-related targets were obtained. By querying various databases, 1080 corneal fibrosis-related targets, 915 corneal injury-related targets, and 698 corneal scar-related targets were obtained. These results were then combined, removing duplicates, and 1445 corneal injury-related targets were selected (Figure 1B). A Venn diagram, generated using online Venn software (http://jvenn.toulouse.inra.fr/app/example.html, accessed on 29 August 2023), revealed 50 common intersecting targets (Figure 1C). These common targets were used to create a PPI network (Figure 1D), which was visualized using Cytoscape software (Version 3.7.2) (Figure 1F). The color and size of each node decrease with a decrease in the node’s degree value, transitioning from red (highest) to yellow (lowest). The color and thickness of edges represent the relationships between nodes. To construct the PPI network, core targets with a degree value greater than 10 were selected (Figure 1G). Some of the high-degree core targets included JUN, CASP3, VEGFA, STAT3, TLR4, MMP9, FGF2, KDR, HSP90AA1, and PPARA, among others, with specific degree values mentioned (Figure 1E).

### 2.2. “LBP Components—Key Targets” Network

**Figure 2:**
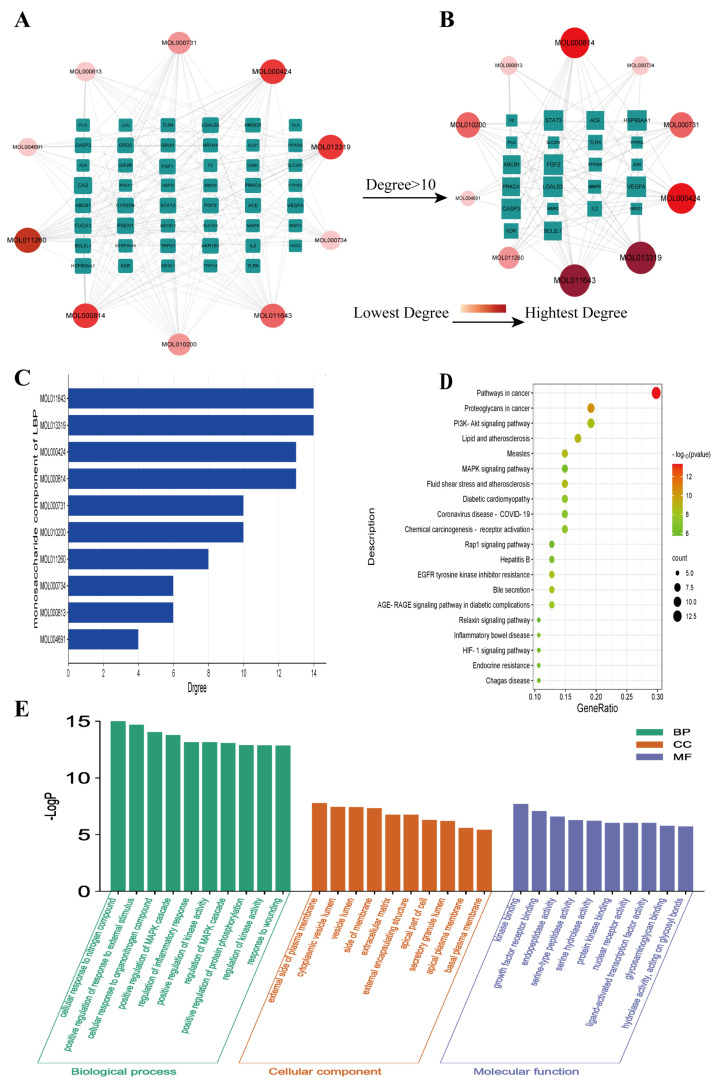
“LBP Component−Key Target” network and enrichment analysis results. (**A**) “Component−Key Target” network; (**B**) “Component−Core Target with Degree Value > 10” network; (**C**) specificity values of LBP monosaccharide components; (**D**) KEGG pathway enrichment analysis; (**E**) GO function enrichment analysis.

Using Cytoscape software (Version 3.7.2), an interaction network was constructed, demonstrating the interactions between LBP’s ten monosaccharide components and 47 potential key targets for corneal injury treatment (Figure 2A). This network comprises 57 nodes and 198 edges, with 47 key targets (green nodes) and 10 active components (ranging from red to pink). The lines between nodes represent the interactions between drug active components and target genes. Further, a network graph was constructed using core targets with a degree value greater than 10 (Figure 2B). The results of this network graph indicate that the active components that interact with over 10 key targets are MOL011643 galacturonic acid, MOL013319 glucuronic acid, MOL000424 rhamnose, and MOL000814 galactose (Figure 2C). These four monosaccharide components are considered the most important and critical components of LBP for corneal injury treatment. The network results illustrate the complexity of interactions between active drug components and therapeutic targets, where one active component can interact with multiple corneal injury-related genes, and multiple active components can also interact with one corneal injury-related gene.

### 2.3. GO Function and KEGG Pathway Enrichment Analysis

**Figure 3:**
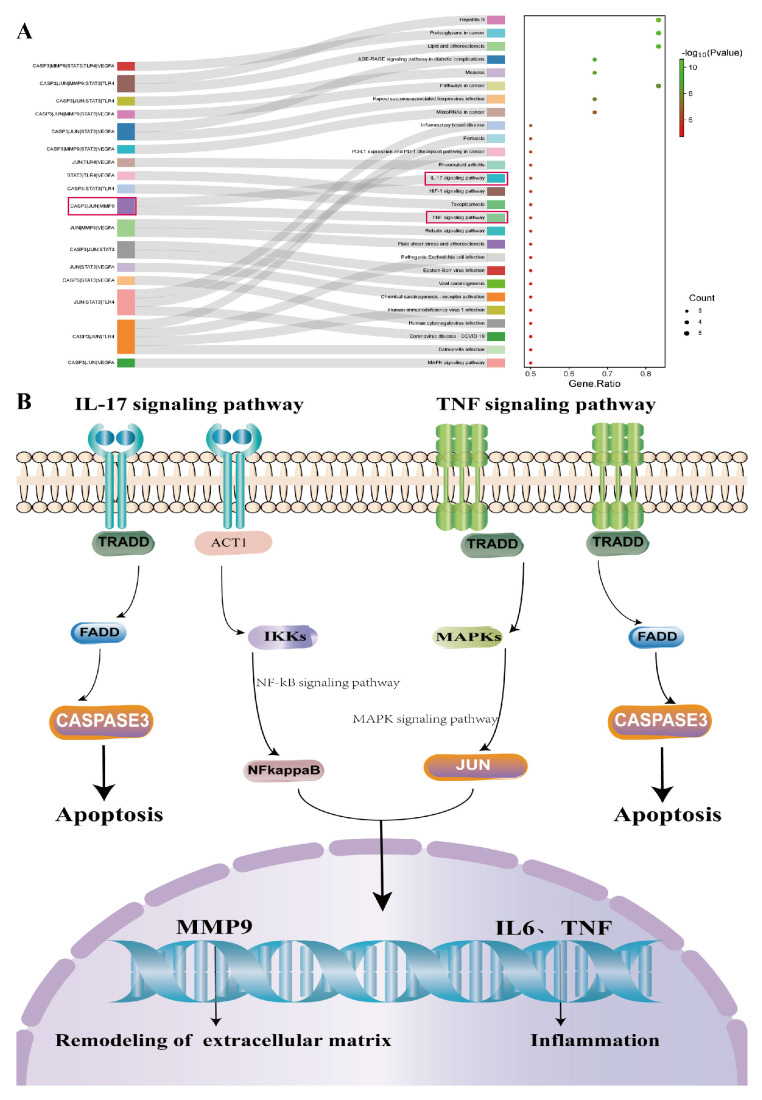
KEGG enrichment analysis of the top 6 core genes. (**A**) KEGG pathway enrichment analysis of the top 6 core genes; (**B**) schematic diagram of the IL17/TNF signaling pathway.

A GO function enrichment analysis was performed for the 47 key targets using the Metascape website, with a significance threshold of *p* < 0.05. A total of 524 biological processes were identified, primarily including the regulation of MAPK cascade, regulation of inflammatory response, and response to injury, among others. Additionally, there were 79 cellular component terms, mainly focused on the outer side of the plasma membrane, cytoplasmic vesicle lumen, extracellular matrix, and 79 molecular function terms involving kinase binding, growth factor receptor binding, and ligand-activated transcription factor activity, among others. The top 10 GO terms from each of the three categories were visualized in a bar chart (Figure 2E). The KEGG pathway enrichment analysis revealed 78 significant signaling pathways (*p* < 0.05), including PI3K-Akt, AGE-RAGE, MAPK, IL-17, TNF, among others. The top 20 pathways were displayed in a bubble chart (Figure 2D). Further enrichment analysis was performed for six core targets with a degree value greater than 20, which resulted in 27 enriched signaling pathways, including IL-17, HIF-1, TNF, MAPK, and others (Figure 3A). Among these, the IL-17 signaling pathway showed a significantly high significance level and included core targets like JUN, CASP3, and MMP9. These core genes were also enriched in the TNF signaling pathway. The IL-17 and TNF signaling pathways are classical inflammatory pathways critical in mediating inflammation, apoptosis, extracellular matrix remodeling, and other reactions that are crucial in corneal injury repair. In summary, the therapeutic effect of LBP on corneal injury repair might be mediated through the IL-17 and TNF signaling pathways via core targets such as JUN, CASP3, and MMP9 (Figure 3B).

### 2.4. Safety Evaluation of LBP for Ocular Application

**Figure 4:**
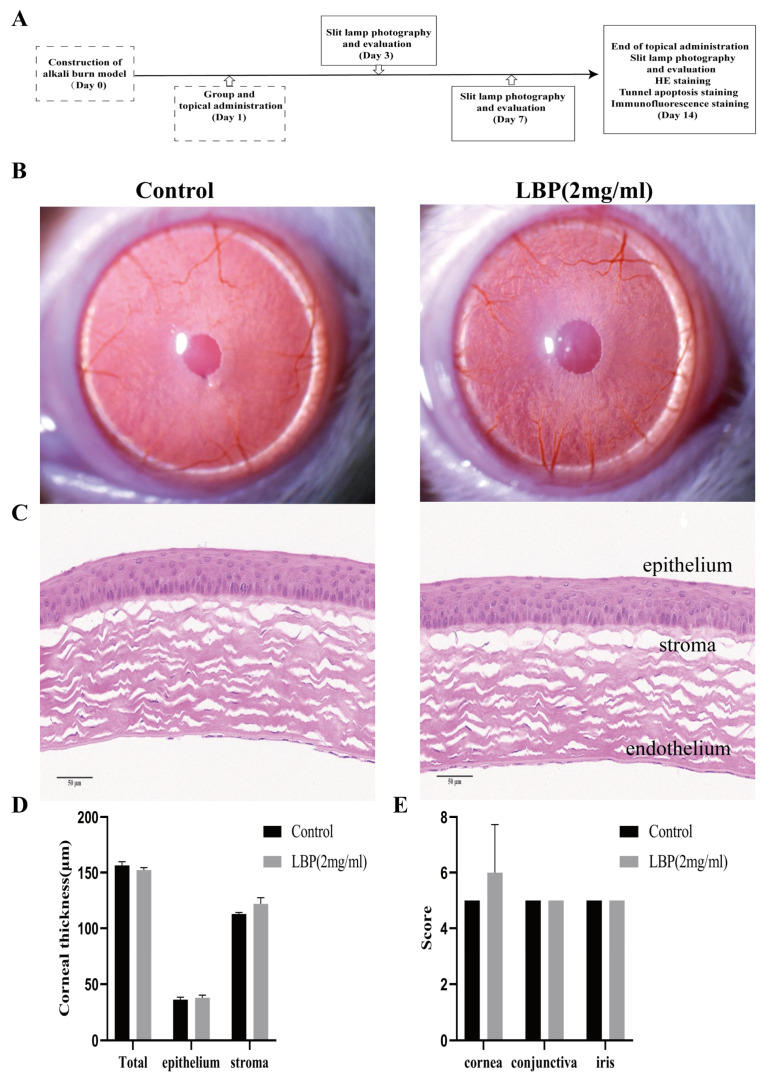
LBP safety evaluation and efficacy experiment design. (**A**) Explanation of the efficacy trial, on day 0, a rat corneal alkali burn model was established, and observations and assessments were made using a slit lamp on days 3, 7, and 14. Corneal HE, TUNEL, and IF staining analyses were performed on day 14; (**B**) slit lamp observations of the cornea in the control group and LBP group after 14 days of eye drops; (**C**) rat corneal HE staining results (scale bar size: 50 μm); (**D**) quantification of corneal thickness in the full layer, epithelium, and stroma; (**E**) statistical chart of ocular irritation response scores for the cornea, conjunctiva, and iris.

After instillation of physiological saline and LBP eye drops, rats exhibited varying degrees of blinking movements, which disappeared within 1 min. There were no head shaking, eye scratching, or other abnormal behaviors observed. Observations were made using a slit lamp on day 14 after LBP eye drop administration, and no significant changes or abnormalities were detected in the eyes of both groups of rats (Figure 4B). The corneas of the rats remained transparent with no opacities, defects, or conjunctival redness or swelling. Comparative assessment of corneal, irideal, and conjunctival irritation reaction scores showed no statistically significant differences between the two groups (Figure 4E). Hematoxylin and eosin (HE) staining results revealed that the corneal epithelium of rats in both groups showed no defects. The stromal layer had regular collagen fiber arrangements, and there was no infiltration of inflammatory cells. The thickness of the corneal epithelium and stromal layer showed no significant differences (Figure 4C,D).

### 2.5. LBP Improves Corneal Tissue Morphology, Reduces Corneal Inflammation, and Decreases Opacity after Alkali Burn Injury

**Figure 5:**
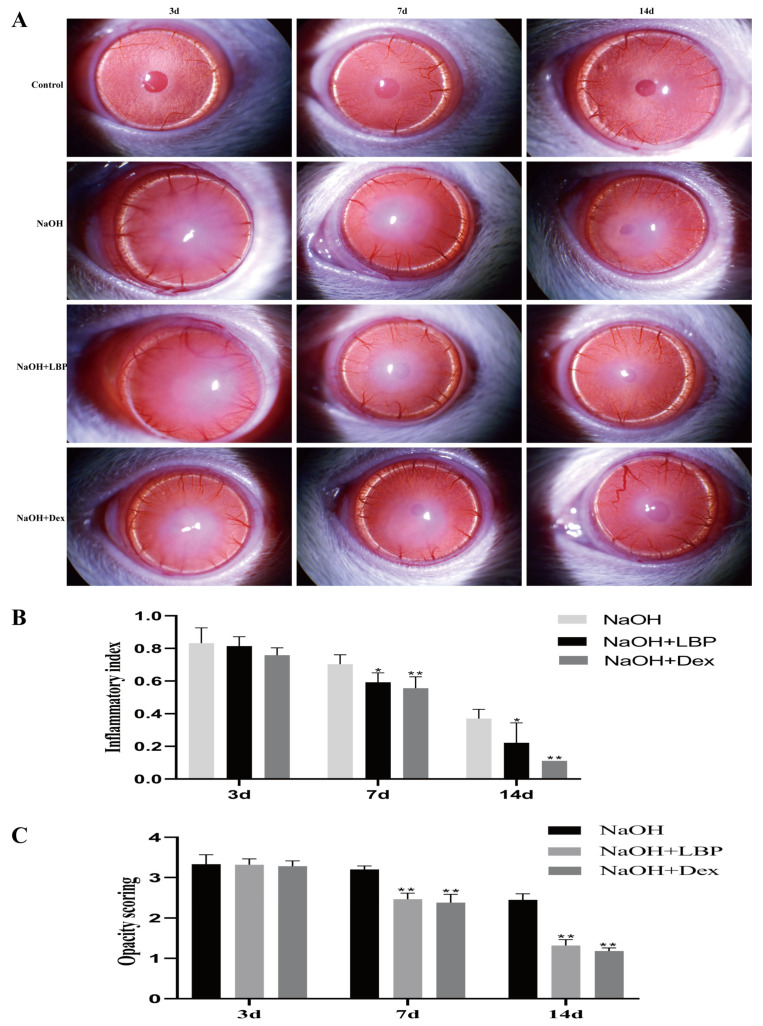
LBP reduces corneal inflammation and opacity after alkali burn. (**A**) Slit lamp observations of rat corneas in each group on days 3, 7, and 14 after alkali burn; (**B**) statistical chart of corneal inflammation index; (**C**) statistical chart of corneal opacity scores. Note: *, ** represent *p* < 0.05, *p* < 0.01 compared to the NaOH group.

Using a slit lamp to observe the corneas of rats in different groups, corneal opacity and inflammation scores were assessed (Figure 5). On day 3, all groups of rats displayed eyelid edema, corneal edema, and moderate to severe opacity. The iris texture was unclear, and the pupils were not visible. On day 7, the corneas in the model group showed more significant edema and opacity, while the corneas in the LBP and Dex intervention groups exhibited slight edema, with lower corneal opacity and inflammation scores compared to the NaOH group (*p* < 0.01). By day 14, the eyelid edema and edema of the model group had improved, while the corneas in the LBP and Dex intervention groups had almost returned to transparency.

**Figure 6:**
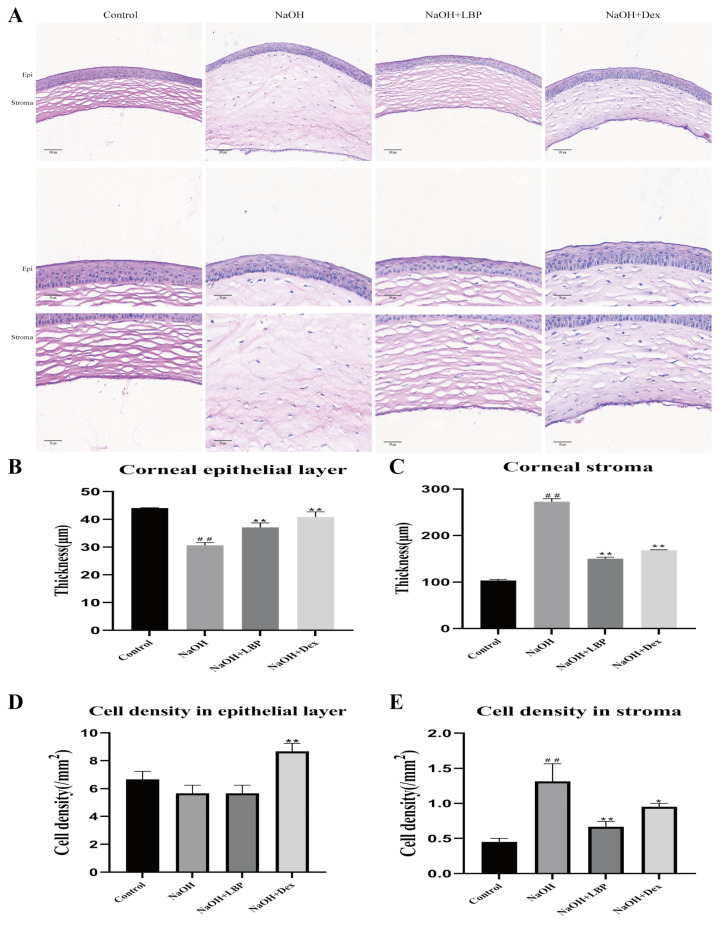
LBP improves corneal tissue morphology and inflammatory cell infiltration after alkali burn. (**A**) Representative images of HE staining (corneal full layer scale bar size: 100 μm, epithelium, and stroma layer images scale bar size: 50 μm); (**B**,**C**) statistical chart of corneal epithelial and stromal thickness; (**D**,**E**) statistical chart of corneal epithelial and stromal cell density. Note: *, ** represent *p* < 0.05, *p* < 0.01 compared to the NaOH group; ## represents *p* < 0.01 compared to the Control group.

In normal rats, the corneal structure was clear, consisting of the epithelial layer, stromal layer, and endothelial layer. The epithelial layer had approximately 6–8 cell layers with a regular morphology, and the collagen fibers in the stromal layer were well-arranged (Figure 6). After alkali burn injury for 14 days, the corneas in the model group exhibited thickening and swelling, significant thinning of the epithelial layer, and a substantial increase in stromal layer thickness and inflammatory cell density (*p* < 0.01). Treatment with LBP following alkali burn injury resulted in slight thickening of the epithelial layer (*p* < 0.05), a noticeable reduction in stromal layer thickness, and a significant decrease in inflammatory cell density (*p* < 0.01). The Dex intervention group showed a significant increase in epithelial thickness (*p* < 0.01), an obvious increase in epithelial cell density (*p* < 0.05), an increase in stromal layer thickness, and an increase in inflammatory cell density compared to the NaOH group (*p* < 0.01, *p* < 0.05). LBP intervention for corneal injury repair led to corneal stromal structure restoration closer to that of the control group compared to the Dex group, while the Dex-treated group exhibited a higher increase in epithelial cell density than the LBP-treated group.

### 2.6. LBP Reduces Apoptosis of Corneal Cells after Alkali Burn Injury

**Figure 7:**
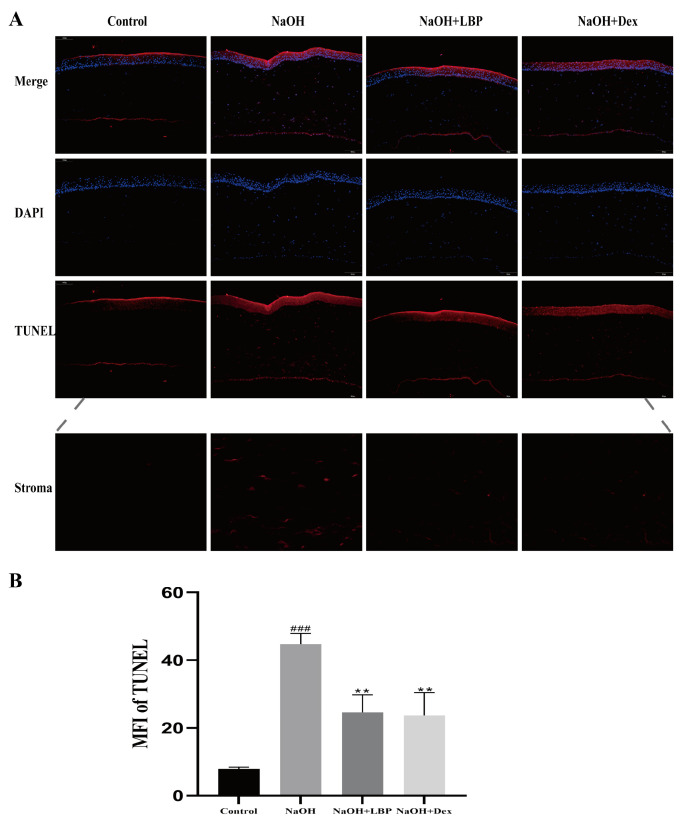
LBP reduces apoptosis of corneal cells after alkali burn. (**A**) TUNEL apoptosis staining results of rat corneas (scale bar size: 100μm). Red fluorescence represents TUNEL positive cells, blue fluorescence is the nucleus stained with DAPI; (**B**) statistical chart of TUNEL mean fluorescence intensity (MFI). Note: ** represent *p* < 0.01 compared to the NaOH group; ### represents *p* < 0.001 compared to the Control group.

TUNEL apoptosis staining of corneal cells in rats showed that, compared to the control group, the NaOH group had more TUNEL-positive cells, while the administration of LBP and Dex resulted in a decrease in red fluorescence signals (Figure 7A). Comparative analysis of the mean fluorescence intensity (MFI) of TUNEL among the groups indicated that alkali burn injury increased corneal cell apoptosis, and the administration of LBP and Dex reduced cell apoptosis (Figure 7B).

### 2.7. LBP Suppresses the Expression of Key Proteins Related to Corneal Fibrosis after Alkali Burn Injury

**Figure 8:**
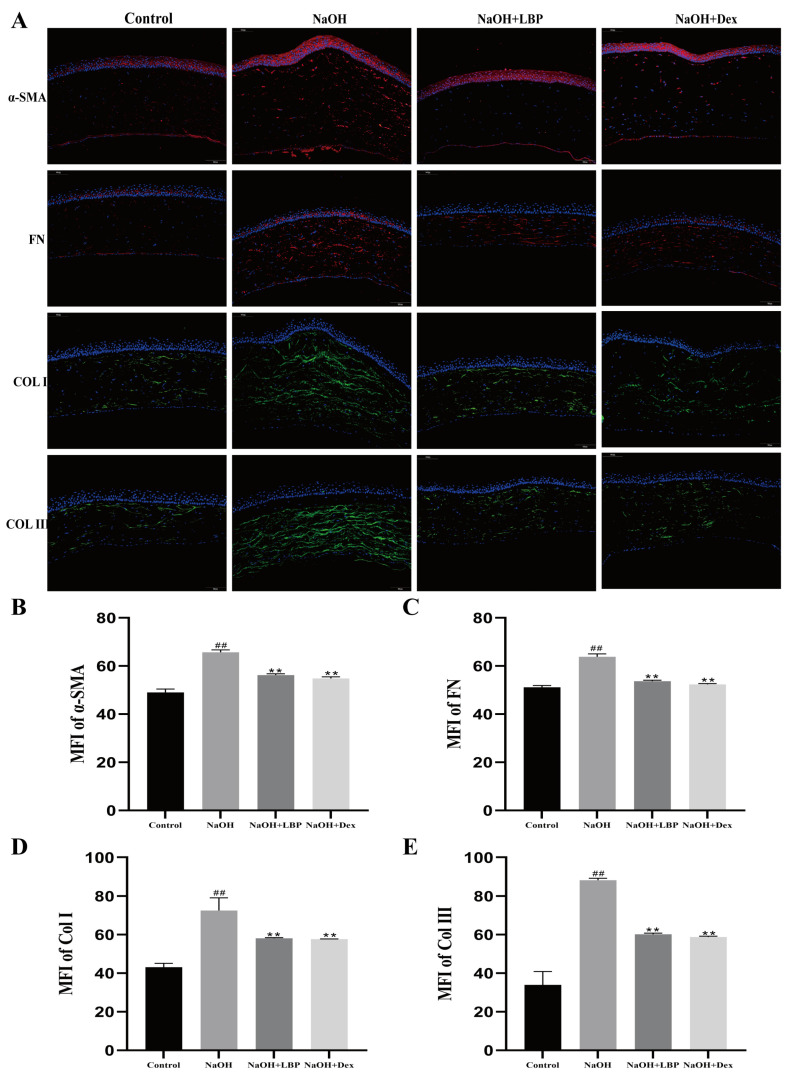
LBP suppresses the expression of fibrosis-related proteins after corneal alkali burn. (**A**) Immunofluorescence staining of rat corneal α-SMA, FN, Col I, Col III (scale bar size: 100 μm), where α-SMA and FN are in red fluorescence, and Col I and Col III are in green fluorescence, with cell nuclei stained blue with DAPI; (**B**–**E**) statistical chart of α-SMA, FN, Col I, Col III mean fluorescence intensity (MFI). Note: ** represent *p* < 0.01 compared to the NaOH group; ## represent *p* < 0.01 compared to the Control group.

The proteins α-SMA, FN, Col I, and Col III are crucial in the process of corneal injury repair. Overexpression of these proteins can lead to decreased corneal transparency and the formation of fibrotic scar tissue. Therefore, the expression of fibrotic-related proteins after alkali burn injury was evaluated. Immunofluorescence (IF) staining of corneal tissues from rats on day 14 after injury was performed. The results (Figure 8A) showed that, compared to the Control group, the NaOH group exhibited increased fluorescence expression of the four proteins in the stromal layer. However, LBP and Dex interventions were able to suppress the expression of these key proteins (Figure 8B–E).

### 2.8. LBP’s Impact on the Viability of HCEC Cells Induced by H_2_O_2_

**Figure 9:**
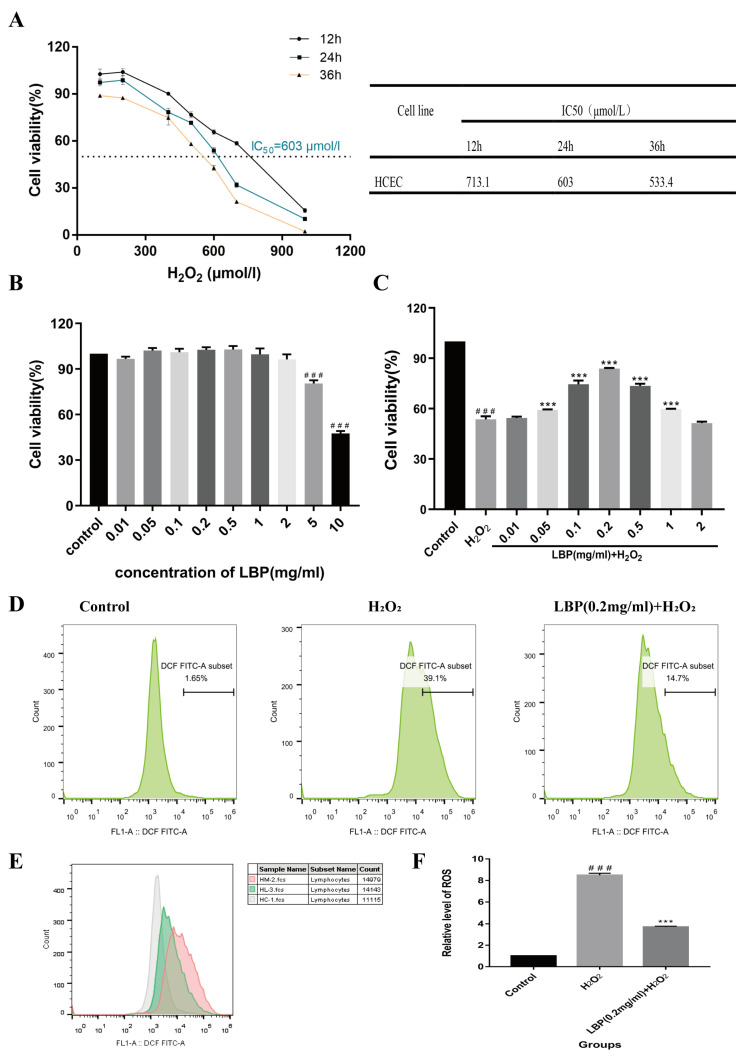
Effects of LBP on H_2_O_2_-induced HCEC cell viability and ROS levels. (**A**) Effects of H_2_O_2_ on HCEC cell viability and IC50 values at different time points; (**B**) effects of different concentrations of LBP on HCEC cell viability; (**C**) effects of different concentrations of LBP on H_2_O_2_ -induced HCEC cell viability; (**D**) flow cytometry of ROS for each group; (**E**) overlapping flow cytometry graphs for ROS (gray is the control group, pink is the H_2_O_2_ group, and green is the LBP + H_2_O_2_ group); (**F**) statistical chart of relative ROS levels. Note: *** represent *p* < 0.001 compared to the H_2_O_2_ group; ### represents *p* < 0.001 compared to the Control group.

Results from the CCK-8 experiment indicated that at 12, 24, and 36 h, cell viability gradually decreased with increasing H_2_O_2_ concentration. At 24 h, with an H_2_O_2_ concentration of 603 μmol/L, cell viability was reduced to around 50%. This time and concentration were chosen for inducing the HCEC cell oxidative stress injury model (Figure 9A). We also tested whether different concentrations of LBP (0.01, 0.05, 0.1, 0.2, 0.5, 1, 2, 5, 10 mg/mL) alone had adverse effects on HCEC cells (Figure 9B). Compared with the control group, LBP concentration in the range of 0.01 to 2 mg/mL had no effect on HCEC cell activity. Drug concentrations in this range were selected for follow-up experiments. LBP concentrations ranging from 0.05 mg/mL to 1 mg/mL could inhibit the damage caused by H_2_O_2_ on HCEC cells and promote cell proliferation (Figure 9C). The cell activity was the highest in the 0.2 mg/mL LBP concentration group, so 0.2 mg/mL LBP concentration was selected to continue the follow-up experiment.

### 2.9. LBP Reduces HCEC Intracellular ROS Levels Induced by H_2_O_2_

The results of the ROS experiment (Figure 9D–F) showed that after 24 h of incubation with 603 μM H_2_O_2_, HCEC cells exposed to H_2_O_2_ accumulated significantly higher levels of intracellular ROS compared to the control group (*p* < 0.001). This suggests that HCEC was in a state of metabolic imbalance, with ROS accumulation exceeding clearance. LBP intervention alleviated this imbalance, reducing intracellular ROS levels (*p* < 0.001).

### 2.10. Effects of LBP on the Cell Cycle of HCEC Induced by H_2_O_2_

**Figure 10:**
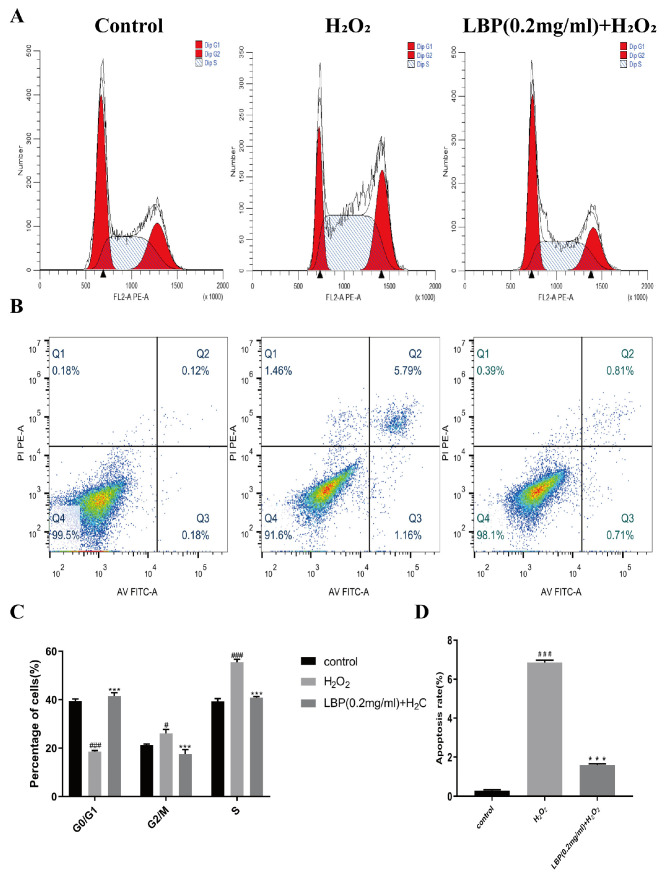
Effects of LBP on H_2_O_2_ -induced HCEC cell cycle and apoptosis. (**A**,**C**) Cell cycle results and statistical charts; (**B**,**D**) apoptosis results and statistical charts. Note: *** represents *p* < 0.001 compared to the H_2_O_2_ group; #, ### represent *p* < 0.05, *p* < 0.001 compared to the Control group.

The cell cycle of HCEC cells was assessed after 24 h of co-intervention with LBP and H_2_O_2_. The results (Figure 10A,C) indicated that, compared to the control group, the H_2_O_2_ group had a decreased proportion of cells in the G0/G1 phase (*p* < 0.001) and increased proportions of cells in the G2/M (*p* < 0.05) and S phases (*p* < 0.001). In comparison to the H_2_O_2_ group, the LBP group had an increased proportion of cells in the G0/G1 phase and decreased proportions of cells in the G2/M and S phases (*p* < 0.001). This suggests that H_2_O_2_ arrested the HCEC cell cycle in the G2/M and S phases, and LBP alleviated this effect, promoting cell cycle progression from the G2/M and S phases to the G1/G0 phase, indicating that LBP facilitates the overall cell cycle.

### 2.11. LBP Reduces Apoptosis of HCEC Cells Induced by H_2_O_2_

After 24 h of co-intervention with LBP and H_2_O_2_, the apoptosis of HCEC cells was assessed. The results (Figure 10B,D) showed that oxidative stress induced by H_2_O_2_ significantly increased the number of apoptotic cells, whereas the LBP group exhibited a reduced apoptosis rate compared to the H_2_O_2_ group (*p* < 0.001).

### 2.12. LBP Enhances HCEC Migration and Healing Capacity Induced by H_2_O_2_

**Figure 11:**
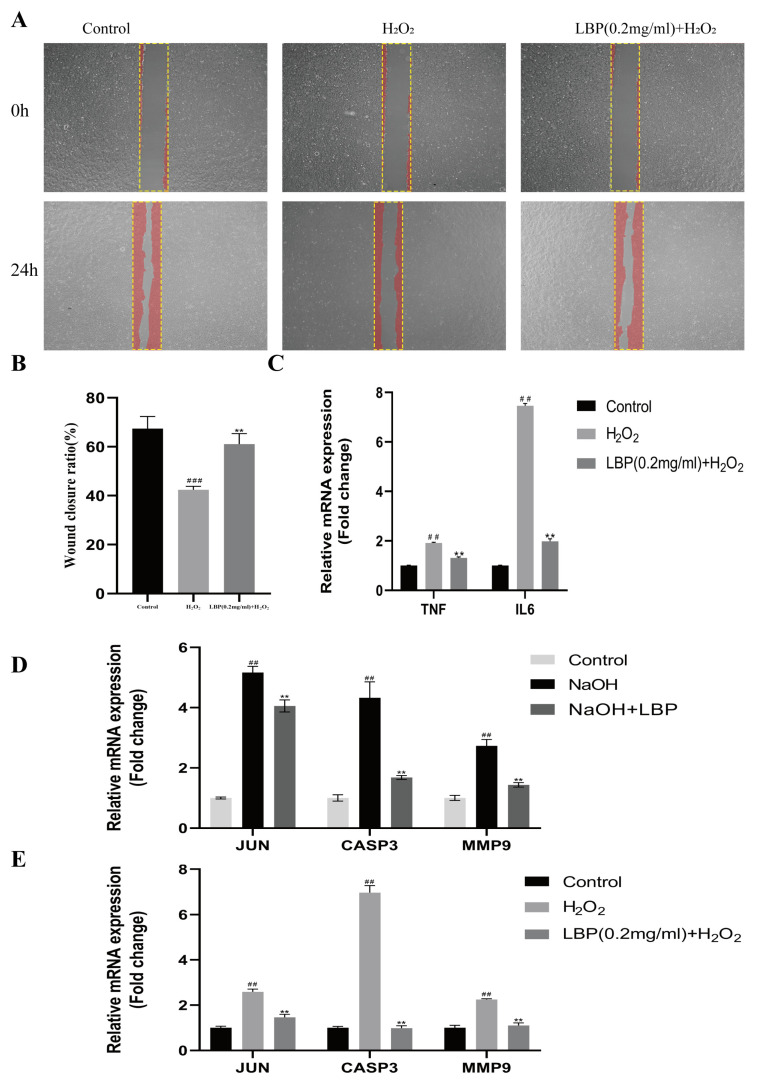
Effects of LBP on h_2_O_2_-induced HCEC cell migration, inflammation, and related gene expression levels. (**A**,**B**) Scratch results and statistical charts (20×), The yellow rectangle is the scratched area and the red area is the cell; (**C**) qRT-PCR detection of TNF-α and IL-6 expression levels in HCEC cells; (**D**) qRT-PCR detection of rat corneal JUN, CASP3, and MMP9 mRNA levels; (**E**) qRT-PCR detection of HCEC cell JUN, CASP3, and MMP9 mRNA levels. Note: ** represent *p* < 0.01 compared to the H_2_O_2_/NaOH group; ##, ### represents *p* < 0.01, *p* < 0.001 compared to the Control group.

Results from a scratch test (Figure 11A,B) demonstrated that the H_2_O_2_ group, compared to the normal group, inhibited the migration and healing capacity of HCEC cells (*p* < 0.01). However, LBP intervention alleviated the inhibitory effect of H_2_O_2_ on cell migration (*p* < 0.01).

### 2.13. LBP Reduces Inflammation in HCEC Cells Induced by H_2_O_2_

To investigate the effect of LBP on inflammation in HCEC cells induced by H_2_O_2_, we used qRT-PCR to assess the expression levels of the TNF-α and IL-6 genes in the cells. Compared to the control group, the H_2_O_2_ group showed an increase in the expression levels of IL6 and TNF-α mRNA (*p* < 0.01). In comparison to the H_2_O_2_ group, LBP intervention significantly reduced the mRNA expression levels of IL6 and TNF-*α* in HCEC cells (*p* < 0.01) (Figure 11C).

### 2.14. LBP’s Effect on the mRNA Levels of Genes Related to Alkali Burned Corneas and H_2_O_2_-Induced HCEC

JUN, CASP3, and MMP9 were identified as core target genes for LBP treatment of corneal injuries in the first part of this study. LBP improves corneal inflammation, cell apoptosis, and extracellular matrix remodeling by targeting these genes. Therefore, we assessed the impact of LBP on the expression levels of these factors in alkali-burned corneas and H_2_O_2_ -induced HCEC cells to validate the network pharmacology predictions. Compared to the control group (Figure 11D), the corneas of the NaOH group showed increased expression of JUN, CASP3, and MMP9 (*p* < 0.01). LBP intervention significantly reduced the mRNA expression levels of JUN, CASP3, and MMP9 in rat corneal tissues (*p* < 0.01). In comparison to the control group, H_2_O_2_-exposed cells exhibited increased expression of JUN, CASP3, and MMP9 (*p* < 0.01), while LBP intervention resulted in a marked reduction in the mRNA expression levels of JUN, CASP3, and MMP9 (*p* < 0.01) (Figure 11E).

## 3. Discussion

Corneal injury repair is a complex process involving oxidative stress, inflammation, cell apoptosis, migration, proliferation, differentiation, and remodeling of the extracellular matrix. LBP are the primary bioactive constituents of goji berries, renowned for their diverse biological activities, such as inhibiting oxidative stress, anti-inflammatory responses, modulating lipid metabolism, and suppressing fibrosis. LBP has been shown to prevent and protect against a variety of ocular diseases. Previous research [1,4,17] indicates that LBP intervention can inhibit HCEC apoptosis, promote proliferation, and reduce the expression of pro-fibrotic proteins and inflammatory cytokines in a model of corneal matrix injury. However, the precise mechanisms remain obscure. In this study, we initially employed network pharmacology techniques to investigate the potential mechanisms by which LBP ameliorates corneal injury. By constructing in vivo and in vitro corneal injury models, we subsequently validated the protective effects of LBP and its associated mechanisms. This provides new insights and directions for LBP corneal injury treatment research.

This study identified ten monosaccharide components of LBP that are efficacious for corneal injury treatment. The most significant effects were exhibited by galacturonic acid, glucuronic acid, rhamnose, and galactose. These four monosaccharide components are regarded as the most vital and essential for the treatment of corneal injury. Galacturonic acid is a functional monosaccharide, and its concentration is a significant factor in LBP activity [15]. Bao Le and others [18] discovered in their investigation of soybean residue polysaccharides that this polysaccharide is primarily composed of galacturonic acid, xylose, and arabinose. Experiments on cells in vitro revealed its potent anti-inflammatory properties, as it inhibited the production of nitric oxide, tumor necrosis factor (TNF)-α, interleukin (IL)-1, and IL-6. Sylla Balla and colleagues [19] discovered that xylose could reduce the intestinal epithelial cell inflammation caused by lipopolysaccharide. Xylose may accomplish this by inhibiting the NF-B pathway and decreasing the NLRP3 inflammasome, as well as other signaling pathways. D-xylosyl is the predominant form of xylose in the human body. After a series of derivations, xylose can produce the small molecule xylose-binding lectin 3 which, when expressed in cells, can regulate cell proliferation, differentiation, and migration. If secreted outside the cell, it can bind directly to lipopolysaccharide, inhibit the production of pro-inflammatory cytokines, and exert an anti-inflammatory effect. In conclusion, the therapeutic effect of LBP on corneal injury may result from its monosaccharide components’ regulation of cell proliferation, apoptosis, antioxidant, and anti-inflammatory effects.

Through KEGG pathway enrichment analysis of the six primary target genes with degrees greater than 20, we discovered that a total of 27 signaling pathways, including IL-17, HIF-1, TNF, and MAPK, were enriched, with the IL-17 signaling pathway being the most significant. Significant enrichment of core target genes, including JUN, CASP3, and MMP9, was observed along this pathway. In distinct cell types, JUN plays different roles in inducing and inhibiting apoptosis [20]. CASP3, also known as caspase-3, is one of the key mediators of cell apoptosis, and its activation can ultimately result in apoptosis [21]. MMP9, or matrix metalloproteinase-9, is the predominant matrix metalloproteinase synthesized and secreted by basal corneal epithelial cells. After corneal injury, its expression increases, and it predominantly participates in corneal basement membrane degradation and ECM remodeling. It inhibits corneal epithelial regeneration and promotes pathological ulceration and perforation [22]. The “component-target” network demonstrates that these three core genes interact with each of LBP’s ten monosaccharide components. The TNF signaling pathway is also enriched for these three essential genes. The IL-17 and TNF signaling pathways are classical inflammatory pathways that mediate diverse reactions, such as inflammation, apoptosis, and extracellular matrix remodeling, which are essential for corneal injury repair. Inducing numerous inflammatory and chemotactic factors, the IL-17 signaling pathway can mediate a series of inflammatory responses and tissue remodeling. The TNF signaling pathway not only mediates inflammatory responses and ECM remodeling, but also promotes cell apoptosis [23]. Experiments in vitro have demonstrated that IL17 and TNF are highly expressed in corneal epithelial cells under conditions of elevated osmotic stress [24]. Du [1] discovered that LBP can inhibit UVB-induced HCEC apoptosis by inhibiting caspase-3 upregulation. Wong [17] pretreated mouse corneas with 2 mg/mL of LBP for 7 days before causing corneal injury with NaOH. The findings demonstrated that LBP intervention could promote corneal epithelial growth and reduce collagen structural damage following injury. Through in vitro cell experiments, this group [4] also demonstrated that LBP could reduce pro-fibrotic proteins and pro-inflammatory cytokines in corneal injury and reduce fibrotic scarring. In conclusion, the ten monosaccharide components of LBP may exert their therapeutic effects on corneal injury via the IL17 and TNF signaling pathways mediated by core genes such as JUN, CASP3, and MMP9, influencing inflammation, apoptosis, and extracellular matrix remodeling responses.

Prior to the clinical implementation of a new drug or drug formulation, it is essential to investigate its toxicity. Typically, drug-induced toxicity in the body begins with functional changes in the organism which, if severe, can result in microscopic morphological changes. In a mouse model of dry eye disease (DED), Qin [25] confirmed the safety and efficacy of LBP eye drops. None of the concentrations of LBP eye drops utilized demonstrated significant eye irritation. In our study, we observed rodents blinking their eyes after receiving LBP eye drops, but this behavior was also observed in the saline group, most likely due to the lower storage temperature of the medication. In addition, this reaction resolved rapidly, and after 14 days of LBP eye drops, there were no statistically significant differences between the two groups’ eye irritation response scores. The results of Hematoxylin and Eosin (HE) staining demonstrated that there were no anomalies in the corneal epithelial cells of either group of rats, and that the stromal fiber arrangement was regular and devoid of inflammatory cell infiltration. These findings demonstrate that the prepared LBP eye drops are safe for short-term, local use in ocular experiments, establishing the groundwork for future research. Numerous studies have demonstrated the protective effect of LBP against corneal injury. LBP intervention can substantially improve the clinical symptoms of dry eye syndrome in mice [17], promote corneal epithelial growth, and cell experiments corroborate that LBP can reduce pro-inflammatory cytokines [4,17]. LBP can also prevent UVB-induced apoptosis in rat corneal epithelial cells [1]. In this section of the study, we discovered that LBP treatment stimulated corneal epithelial growth after alkaline injury, decreased corneal opacity, and decreased the infiltration of inflammatory cells into the corneal stroma. In the alkali burn model, the restoration of the corneal stromal layer structure following LBP intervention was closer to that of the control group than that of the Dex (dexamethasone) group. However, Dex treatment resulted in a greater increase in cell density in the corneal epithelial layer than LBP treatment. The results of TUNEL immunofluorescence staining demonstrated that both LBP and Dex interventions decreased the number of apoptotic cells. Under typical conditions, the collagen fibers in the corneal stromal layer are arranged and distributed in a uniform manner. Chemical injuries to the cornea can result in the loss of corneal epithelial layers, stromal edema [26], excessive deposition of the ECM, and decreased degradation, leading to late-stage infection and the formation of fibrotic scars. This can impair a patient’s eyesight. α-SMA, FN, Col I, and Col III are essential proteins in fibrosis. Immunofluorescence staining results indicated that both LBP and Dex could inhibit the expression of important corneal fibrotic proteins following alkali injury. These results corroborate that LBP has a protective effect on corneal injury and can improve the degree of fibrosis during the corneal injury repair process. LBP’s therapeutic effect is not substantially different from that of Dex, and it overcomes Dex’s disadvantage of a short application period, allowing for a longer application period and greater efficacy.

Previous research [1] has demonstrated that HCEC treated with 0.05–1 mg/mL LBP can prevent the UVB-induced decrease in cell viability. In our study, LBP concentrations between 0.05 mg/mL and 1 mg/mL inhibited H_2_O_2_-induced cell injury and promoted cell proliferation in HCEC cells. The maximum cell activity was observed in the 0.2 mg/mL LBP group. H_2_O_2_ inhibited HCEC proliferation and migration, which resulted in a cell cycle arrest in the G2/M phase and a decrease in the G0/G1 phase, which inhibited cell division and decreased cell proliferation. After LBP intervention, however, the G2/M phase cell cycle arrest caused by oxidative stress-induced HCEC damage was reduced, promoting cell division and transition from G2/M to G1/G0 phase, facilitating cell proliferation and migration, and reducing cell damage, thereby restoring normal physiological function. These studies indicate that LBP in the appropriate concentration range has no substantial impact or toxic side effects on the cells themselves, and can enhance epithelial cell proliferation and migration following corneal epithelial injury. It is anticipated to be a safe and efficacious corneal ophthalmic formulation. The detection of reactive oxygen species (ROS) revealed a significant increase in intracellular ROS levels as a result of oxidative stress damage, placing HCEC in a metabolic state of imbalance with ROS accumulation exceeding clearance. ROS is intimately associated with the mitochondrial pathway of apoptosis, where mitochondria are the primary source of ROS production [27]. The accumulation of ROS can alter mitochondrial membrane permeability, causing apoptotic factors such as Cytochrome C to be released from mitochondria into the cytoplasm, thereby initiating the caspase cascade and activating the final apoptotic executor caspase-3 [28], resulting in an increase in cell apoptosis [29,30], confirming this theory. Flow cytometry apoptosis assay results confirmed that oxidative stress injury increased the number of apoptotic HCEC significantly. LBP intervention ameliorated oxidative stress-induced HCEC injury by decreasing ROS levels in the cells, thereby reducing HCEC cell apoptosis. Oxidative stress is associated with inflammation [31,32,33]; for example, in dry eye syndrome, oxidative stress resulting from an imbalance of reactive oxygen species (ROS) is the primary cause, and inflammatory reactions are a major source of ROS [34,35]. Related research has confirmed the relationship between oxidative stress and inflammation [36,37], and certain types of inflammation can contribute to the progression of disease through the excessive production of reactive oxygen species (ROS) and other oxidants, and a decrease in antioxidant levels [38]. In eye diseases, the decrease in lactoferrin levels in the tear film, an antioxidant that bonds with free iron to reduce ROS production, causes an increase in infections and inflammatory responses. Overproduction of reactive oxygen species (ROS) in eye surface cells can activate inflammatory signaling pathways to promote inflammation, leading to an increase in pro-oxidant-sensitive inflammatory cytokines such as TNF-α, IL-6, IL-8, etc. [39]. In HCEC, oxidative stress substantially increased the levels of reactive oxygen species (ROS) and the expression of inflammatory factors IL6 and TNF-α, according to our research. However, after LBP intervention, both ROS levels and IL6 and TNF-α gene expression were significantly reduced. This suggests that LBP can inhibit the production of reactive oxygen species (ROS) in HCEC, thereby reducing the inflammatory response.

In summary, by examining the gene expression levels of the predicted core targets (JUN, CASP3, and MMP9) in the rat cornea and HCEC after LBP intervention, we found that LBP treatment significantly reduces the mRNA levels of JUN, CASP3, and MMP9 in both in vivo and in vitro corneal injury models. These three factors are all enriched in the TNF and IL17 signaling pathways. Therefore, we hypothesize that LBP may mitigate corneal inflammation, cell apoptosis, and extracellular matrix remodeling after corneal injury by inhibiting the relevant signaling pathways mediated by JUN, CASP3, and MMP9.

## 4. Materials and Methods

The databases and software used for network pharmacology prediction analysis are detailed in Supplementary Table S1.

### 4.1. Screening Targets for LBP-Mediated Corneal Injury Repair and Constructing Protein–Protein Interaction (PPI) Network

The monosaccharide composition information of LBP was obtained from cutting-edge research. Through the TCMSP platform, we searched for the chemical structures, oral bioavailability (OB), and drug-likeness (DL) of individual monosaccharide components of LBP. Furthermore, we retrieved the linear notations of each monosaccharide component from the PubChem database, and predicted the relevant targets of LBP based on these linear notations in the SwissTarget database. Using five databases (GeneCards, OMIM, DrugBank, PharmGKB, and DisGeNET), we conducted keyword searches with terms such as “corneal fibrosis, corneal injury, corneal scar” to obtain genes related to corneal injury diseases. The intersection of disease-related targets and LBP active ingredient targets was obtained using Venn diagram online software (http://jvenn.toulouse.inra.fr/app/example.html, accessed on 29 August 2023) to identify potential targets for LBP in the treatment of corneal injury diseases. The PPI network diagram was created using the STRING 11.5 online database. The topological parameters of the network were calculated using Cytoscape (Version 3.7.2) software, and targets with high degree values were selected for subsequent analysis.

### 4.2. Construction and Analysis of “Drug Components—Key Targets” Network

LBP monosaccharide components, intersecting targets, and other data were imported into Cytoscape software (Version 3.7.2) for visual analysis and network creation. Network topology analysis tools were used to calculate the degree of each node, which represents the number of nodes directly connected to it. A higher degree value indicates a more critical role of that node in the network.

### 4.3. KEGG Pathway and GO Function Enrichment Analysis

Enrichment analysis of the obtained key targets was conducted using the Metascape database. GO analysis was performed in three modules: biological processes (BP), molecular functions (MF), and cellular components (CC), to understand the biological processes, molecular functions, and cellular components involved with the target genes in vivo. KEGG pathway enrichment analysis was carried out to identify the primary signaling pathways associated with LBP’s treatment of corneal injury diseases, with a significance level set at *p* < 0.05. The results of both enrichment analyses were visualized as bar charts and bubble charts using a bioinformatics platform.

### 4.4. Animal Experiment Grouping and Topical Administration

LBP solution was prepared by accurately weighing LBP powder (SP9311, Solarbio, Beijing, China) in a clean bench and dissolving it in physiological saline to achieve a final concentration of 2 mg/mL. The solution was then sterilized by filtering through a 0.22 μm sterile micropore membrane and stored in a 4 °C refrigerator.

Healthy male SD rats provided by the Ningxia Medical University Animal Center were examined with a slit lamp to exclude any anterior segment lesions before being randomly assigned to experimental groups. SD rats were placed in 12 h light/12 h dark conditions, free access to food and water in a room controlled by temperature and humidity; rat feed (23103223, Keao Xieli Feed, Beijing, China) raw material composition: corn, soybean meal, fish meal, flour, yeast powder, vegetable oil, salt, vitamins, and mineral elements. A total of 48 rats were used in the experiment, and were divided into 6 groups with 8 rats in each group: (1) Control group (two groups in total): topical administration of saline. (2) LBP group: topical administration of 2 mg/mL LBP solution.(3) NaOH group: topical administration of saline after alkali burn model creation.(4) NaOH + LBP group: topical administration of 2 mg/mL LBP solution after alkali burn model creation. (5) NaOH + Dex group: topical administration of Dex (H20020497, qilu-pharma, Jinan, China) after alkali burn model creation.

The right eye of each rat was used for the experiment, and each group received the corresponding solution in their eyes, four times a day, for a total of 14 days, with each eye closure lasting for more than 10 s after eye drop administration. Schematic representation of the experimental design of the effectiveness of LBP treatment (Figure 4A).

### 4.5. Construction of Rat Corneal Alkali Burn Model

Rats were anesthetized by intraperitoneal injection of a mixture of 90 mg/kg ketamine and 10 mg/kg xylazine (Alfamedic, Singapore). Local anesthesia was administered by instilling lidocaine hydrochloride on the corneal surface, and excess fluid in the conjunctival sac was wiped away with a cotton swab. A unilateral corneal alkali burn model [40,41,42] was established by using a corneal trephine to create a circular filter paper with a diameter of 3 mm, which was then immersed in a 1M NaOH (A620617, Sangon Biotech, Shanghai, China) solution for 5 s. The filter paper was removed, excess alkali was blotted with filter paper, and it was gently applied to the central cornea of the right eye of the rat. After a 30 s burn, the burned area and the conjunctival sac were rinsed with physiological saline for 1 min. A circular gray-white burn spot, approximately 3 mm in diameter, was visible in the central cornea. The success criteria for the model were corneal stromal edema and obvious opacity, with the iris faintly visible.

### 4.6. Slit Lamp Examination

Before and after administration of the corresponding solution, slit lamp examination was conducted to observe and record the cornea, conjunctiva, and iris of the rats. Evaluation was performed based on the modified Draize eye irritation test scoring system, with the scores added up and multiplied by weighting factors to obtain weighted scores for cornea, iris, and conjunctiva. The corneal inflammation index for each group of rats was calculated by summing the scores and dividing by 9, as per the internationally recognized scoring standard [43]. Corneal opacity in rats was scored according to the criteria described in relevant literature [44].

### 4.7. Hematoxylin and Eosin (H&E) Staining

Whole rat eyeballs were fixed in 4% paraformaldehyde overnight at 4 °C. The tissues were dehydrated using an automated dehydrator and then rapidly embedded in a 60 °C paraffin oven. Paraffin blocks were sectioned into 4 μm thick paraffin sections using a rotary microtome, followed by HE staining. Neutral resin was used for mounting the slides. The corneal thickness and corneal cell density were counted and statistically analyzed using ImageJ software (Version 1.46r).

### 4.8. TUNEL Apoptosis Staining and Immunofluorescence Staining

Fixed rat corneal sections were incubated in 4% paraformaldehyde and permeabilized with 0.5% Triton X-100 at room temperature. After blocking with 5% BSA for 60 min, TUNEL apoptosis staining was performed by incubating the sections in TUNEL detection solution (C1090, Beyotime, Shanghai, China) in a light-protected environment for 1 h. Immunofluorescence staining involved incubating the sections with the respective primary antibody at 4 °C overnight, followed by incubation with fluorescent secondary antibody for 1 h. DAPI-containing anti-fluorescence quenching mounting medium (H-1200-10, Vector Laboratories, Newark, CA, USA) was used for nuclear staining. Observations and photography were carried out under a fluorescence microscope. The average fluorescence intensity (MFI) of apoptosis and fibrosis-related proteins was statistically analyzed using ImageJ software.

This section involves antibodies and reagents, including anti-α-SMA (AF1032-100, Affinity, San Francisco, CA, USA), anti-fibronectin (66042-1-Ig-100, Proteintech, Wuhan, China), anti-collagen I (66761-1-Ig-100, Proteintech, China), and anti-collagen III (22734-1-AP-100, Proteintech, CHN) antibodies, fluorescent secondary antibody (111-545-144, Jackson ImmunoResearch Inc., West Grove, PA, USA), and DAPI-containing anti-fluorescence quenching mounting medium(H-1200-10, Vector Laboratories, Newark, CA, USA).

### 4.9. Cell Culture and Reagents

HCEC (342430, BNCC, CHN) was cultured in EMEM medium supplemented with 10% fetal bovine serum (FBS, Gibco, Grand Island, NY, USA) and antibiotics (100 mg/mL streptomycin and 100 U/mL penicillin) at 37 °C in a 5% CO_2_ incubator. LBP powder was dissolved in pre-configured EMEM complete medium to a final concentration of 10 mg/mL. The solution was filtered and sterilized through a 0.22 μm sterile micropore membrane and stored at −20 °C. The mother solution was diluted with EMEM medium to the required concentration before use.

### 4.10. Cell Counting Kit-8 (CCK-8) Assay

Cells were seeded as a suspension (100 μL/well) in a 96-well plate. After 24 h in a cell culture incubator to allow cells to adhere to the well bottom, the old medium was removed, and cells were treated as per the experimental design. After a specified incubation period, 10 μL of CCK-8 reagent (K04, DOJINDO, Kumamoto, Japan) was added to each well. After incubating for 1.5 h, the absorbance (OD) at 450 nm was measured using a microplate reader to calculate cell viability.

### 4.11. Flow Cytometry

HCEC cells were seeded in 6-well plates at a density of 2 × 10^5^ cells/well. Once cell confluence reached 70–80%, the cells were divided into control group, H_2_O_2_ group, and LBP group. The control group was cultured normally without any treatment, and the H_2_O_2_ group was treated with 603 μmol/L H_2_O_2_ (30%, Cas: 7722-84-1, Yantai Shuangshuang, Yantai, China) for 24 h to create an oxidative stress-damaged cell model. The LBP group received 0.2 mg/mL LBP and 603 μmol/L H_2_O_2_ co-treatment for 24 h. Cell ROS levels, cell cycle, and apoptosis rate were determined using the ROS detection kit (KGT010-1, KeyGEN, Nanjing, China), Annexin V-FITC/PI apoptosis detection kit (KGA107, KeyGEN, China), and cell cycle detection kit (KGA512, KeyGEN, China) following the manufacturer’s instructions. BD AccuriTM C6(Becton, Dickinson and Company, Franklin Lakes, NJ, USA) was used for the determination of cell ROS levels, cell cycle, and apoptosis rate.

### 4.12. Scratch Assay

Healthy HCEC cells were plated at a density of 2 × 10^5^ cells/well in a 6-well plate. When cell confluence reached above 80%, a 10 μL pipette tip was used to create a “—” pattern scratch on the cell monolayer. The old culture medium was removed, and cells were treated according to the respective group. After 24 h, the changes in cell scratch were observed under an inverted microscope.

### 4.13. Real-Time Quantitative Polymerase Chain Reaction (qRT-PCR)

Total RNA was extracted from rat corneal and cell samples from each group using Trizol reagent (ThermoFisher, Waltham, MA, USA). The RNA was reverse-transcribed into cDNA using the PrimeScript™ RT reagent Kit with gDNA Eraser (Perfect Real Time) (RR047A, Takara, Shiga, Japan). TB Green^**®**^ Premix Ex Taq™ II (Tli RNaseH Plus) (RR820A, Takara, Japan) was used for subsequent experiments. PCR was carried out for 40 cycles using a fluorescence quantitative PCR instrument (StepOne Software V2.3, ThermoFisher, USA), with GAPDH as the internal reference gene. The relative gene expression of various genes was analyzed using the 2^−ΔΔCT^ method. Specific primer sequences are available in Supplementary Table S2.

### 4.14. Data Analysis

Data from this study are presented as mean ± standard deviation ($\bar{x}$ ± s). Statistical analysis and graph plotting were performed using GraphPad Prism 9.0 software. The differences between groups were analyzed using *t*-tests or one-way analysis of variance (ANOVA) for homogenous variance, with *p* < 0.05 indicating statistically significant differences.

## 5. Conclusions

Through network pharmacology, this article initially hypothesizes the potential and associated mechanisms of LBP in treating corneal injury, providing some data support for further experimental verification. This study utilized network pharmacology analysis and a combination of in vivo and in vitro experiments to confirm that LBP can reduce inflammation, apoptosis, corneal opacity, and fibrosis in rats caused by chemical corneal injury. It also inhibited the decrease in cell survival rate, migration capacity, and increased in apoptosis rate, ROS levels, expression of inflammatory factors caused by oxidative stress injury in HCEC. LBP may protect the cornea by impeding the TNF and IL-17 signaling pathways via JUN, CASP3, and MMP9. These results establish a research foundation and offer novel perspectives on the process of corneal injury repair. It is important to note, however, that the primary objective of this study is to demonstrate the inhibitory effect of LBP on corneal fibrosis via in vivo experiments on rats. Further investigation is warranted to examine the interventional effects of LBP on human corneal stromal cells. Additionally, due to variations in the anatomical structures of the cornea between rats and humans, it may be necessary to employ distinct in vivo experimental models to validate the therapeutic effects and mechanisms of LBP on corneal injuries.

## Figures and Tables

**Table 1:** Related information of monosaccharide composition in LBP.

MOL ID	Molecule Name	Structure	AlogP	OB (%)	DL	PubChem ID	SMILES
MOL010200	arabinose	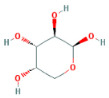	−2	54.12	0.03	439764	C1C(C(C(C(O1)O)O)O)O
MOL000734	glucose	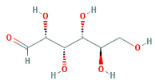	−2.68	24.44	0.03	107526	C(C(C(C(C(C=O)O)O)O)O)O
MOL000814	galactose	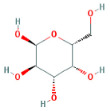	−2.51	47.94	0.04	439357	C(C1C(C(C(C(O1)O)O)O)O)O
MOL000813	mannose	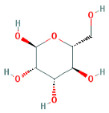	−2.51	20.71	0.04	185698	C(C1C(C(C(C(O1)O)O)O)O)O
MOL000731	xylose	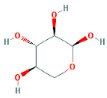	−2	58.74	0.03	6027	C1C(C(C(C(O1)O)O)O)O
MOL000424	rhamnose	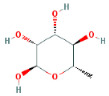	−1.62	50.5	0.04	439710	CC1C(C(C(C(O1)O)O)O)O
MOL013319	Glucuronic acid	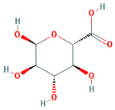	−2.31	63.13	0.06	444791	C1(C(C(OC(C1O)O)C(=O)O)O)O
MOL011643	Galacturonic acid	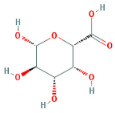	−2.31	40.39	0.06	441476	C1(C(C(OC(C1O)O)C(=O)O)O)O
MOL004691	ribose	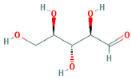	−2.17	40.76	0.02	5311110	C(C(C(C(C=O)O)O)O)O
MOL011260	glucosamine	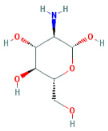	−2.8	50.15	0.04	441477	C(C1C(C(C(C(O1)O)N)O)O)O

## Data Availability

The original contributions presented in the study are included in the article. Further inquiries can be directed to the corresponding authors.

## Supplementary Materials

The following supporting information can be downloaded at: https://www.mdpi.com/article/10.3390/molecules29010049/s1, Table S1: Database and software access urls; Table S2: qRT-PCR Primer sequence.
