# Supplementary material for: Exploring the Role of Lycium barbarum Polysaccharide in Corneal Injury Repair and Investigating the Relevant Mechanisms through In Vivo and In Vitro Experiments
Source: Molecules. 2023 Dec 20;29(1):49. doi: 10.3390/molecules29010049 (PMC10779902; doi:10.3390/molecules29010049)
Supplement: Supplementary file 1 [file molecules-29-00049-s001.zip › Supplementary Materials Table S1.pdf]

Table S1: Database and software access urls

Name	Website
TCMSP	http://tcmspw.com/tcmsp.php
PubChem	https://pubchem.ncbi.nlm.nih.gov/
Swisstarget	http://www.swisstargetprediction.ch/
UniProt	https://www.Uniprot.org/
GeneCards	https://www.genecards.org/
OMIM	https://www.omim.org/
DrugBank	https://go.drugbank.com/
PharmGKB	https://www.pharmgkb.org/
DisGeNET	https://www.disgenet.org/
Venn	http://jvenn.toulouse.inra.fr/app/example.html
STRING11.5	https://string-db.org/cgi/input.pl
Cytoscape	https://cytoscape.org/
Metascape	https://metascape.org/gp/index.html#/main/step1
Bioinformatics	http://www.bioinformatics.com.cn/
