# Supplementary material for: Exploring the Role of Lycium barbarum Polysaccharide in Corneal Injury Repair and Investigating the Relevant Mechanisms through In Vivo and In Vitro Experiments
Source: Molecules. 2023 Dec 20;29(1):49. doi: 10.3390/molecules29010049 (PMC10779902; doi:10.3390/molecules29010049)
Supplement: Supplementary file 1 [file molecules-29-00049-s001.zip › Supplementary Materials Table S2.pdf]

Table S2: qRT-PCR Primer sequence

Genes	Forward	Reverse	Bp
JUN	AGAACTCGGACCTCCTCACCTC	ATGTGCCCGTTGCTGGACTG	97
MMP9	CCTGGTCCTGGTGCTCCTG	GCTGCCTGTCGGTGAGATTG	113
CASP3	TGGAAGCGAATCAATGGACTCTGG	AGACCGAGATGTCATTCCAGTGC	130
TNF	CTCATCTACTCCCAGGTCCTCTTC	CGATGCGGCTGATGGTGTG	82
IL6	AGAGTAGTGAGGAACAAGCCAGAG	CCACACCAAGTTGAGGGAATGAG	105
